# Field based assessment of a tri-axial accelerometers validity to identify steps and reliability to quantify external load

**DOI:** 10.3389/fphys.2022.942954

**Published:** 2022-09-12

**Authors:** Abdulmalek K. Bursais, Jeremy A. Gentles, Naif M. Albujulaya, Michael H. Stone

**Affiliations:** ^1^ Department of Physical Education, College of Education, King Faisal University, Al-Ahsa, Saudi Arabia; ^2^ Center of Excellence for Sport Science and Coach Education, East Tennessee State University, Johnson, TN, United States; ^3^ School of Sport, Exercise and Health Sciences, Loughborough University, Loughborough, Leicestershire, United Kingdom

**Keywords:** wearable technologies, accelerometers, step detection, training load, monitoring, physical activity

## Abstract

**Background:** The monitoring of accelerometry derived load has received increased attention in recent years. However, the ability of such measures to quantify training load during sport-related activities is not well established. Thus, the current study aimed to assess the validity and reliability of tri-axial accelerometers to identify step count and quantify external load during several locomotor conditions including walking, jogging, and running.

**Method:** Thirty physically active college students (height = 176.8 ± 6.1 cm, weight = 82.3 ± 12.8 kg) participated. Acceleration data was collected *via* two tri-axial accelerometers (Device A and B) sampling at 100 Hz, mounted closely together at the xiphoid process. Each participant completed two trials of straight-line walking, jogging, and running on a 20 m course. Device A was used to assess accelerometer validity to identify step count and the test-retest reliability of the instrument to quantify the external load. Device A and Device B were used to assess inter-device reliability. The reliability of accelerometry-derived metrics Impulse Load (IL) and Magnitude g (MAG) were assessed.

**Results:** The instrument demonstrated a positive predictive value (PPV) ranging between 96.98%–99.41% and an agreement ranging between 93.08%–96.29% for step detection during all conditions. Good test-retest reliability was found with a coefficient of variation (CV) <5% for IL and MAG during all locomotor conditions. Good inter-device reliability was also found for all locomotor conditions (IL and MAG CV < 5%).

**Conclusion:** This research indicates that tri-axial accelerometers can be used to identify steps and quantify external load when movement is completed at a range of speeds.

## 1 Introduction

Wearable technologies have become common place in team and individual sports to assess internal and external loads of athletes. These technologies are used to measure various physiological-related variables including heart rate, oxidative muscle metabolism, breathing frequency, skin temperature (Ferrari, Muthalib and Quaresima, 2011; Johnstone et al., 2012a; Johnstone, Ford, Hughes, Watson and Garrett, 2012a; Plews et al., 2013), as well as activity-related variables such as total distance, acceleration, deceleration, and posture (Boyd, Ball and Aughey, 2011; Johnstone, Ford, Hughes, Watson and Garrett, 2012a; Johnstone et al., 2012b; Rawstorn et al., 2014). The estimation of physical workload performed by athletes is of particular importance to many practitioners and coaches. Therefore, devices used to evaluate the physical effort of athletes during practice and competition have become essential components of load monitoring (Akenhead and Nassis, 2016). Accelerometers are one type of wearable technology used to indicate the quantity of mechanical load performed by athletes (Colby et al., 2014), that may be used to improve the ability of practitioners to better manage fatigue and direct adaptation. The role of accelerometers in load monitoring has received increased attention across a number of sports in recent years (Cummins et al., 2013; Chambers et al., 2015). Despite this, the validity and reliability of accelerometers to detect events and quantify external load during sport-related activities is not well established.

Accelerometers are a responsive motion sensor that measure the magnitude of acceleration in one or more axes. Accelerometers are often used to assess the gait of human movement and identify specific types of motion or positions such as locomotor activities and posture (Lugade et al., 2013; Fortune et al., 2014). Events including steps, jumps, kicks, and throws have been identified using accelerometers (Koda et al., 2010; Choukou, Laffaye and Taiar, 2014; Ellens et al., 2017; Pham et al., 2017). However, most studies that have investigated the validity of this technology were conducted in a laboratory setting.

A variety of event-specific algorithms and acceleration thresholds are used in accelerometry based event identification and human movement assessment. For instance, steps are often counted based on toe-off, heel-strike, and/or mid-swing identification with established acceleration thresholds and time between sequential gait events (Salarian et al., 2004). Algorithms using vertical acceleration (Din, Godfrey and Rochester, 2016) or anterior-posterior acceleration (Micó-Amigo et al., 2016), have also been validated to identify step count when a triaxial accelerometer was attached on the lower back, and lower back or heels, respectively. Pham et al. (2017) provides an example of this, finding that in a home-like environment, accelerometry data could be used to detect steps that included turning events with an accuracy of 88% and positive predictive value of 94%, while steps without turning events were detected with 91% accuracy and 98% positive predictive value when a triaxial accelerometer was attached on the lower back. Moreover, to validate step count identification using accelerometers, Fortune et al. (2014) assessed a custom-designed activity monitoring system (AMS) that consisted of four accelerometers positioned at the waist, right thigh, and bilateral ankles. Three different commercial accelerometers were also attached at the ankle, waist, and wrist. The authors conclude that the AMS algorithm could identify steps at a higher median agreement and smaller interquartile range (92% and 8%) than the commercial accelerometers located at the ankle (92% and 36%), waist (93% and 22%), wrist (33% and 35%) when dynamic activities at velocity ranged from 0.1 to 4.8 m/s were performed. This suggests that the algorithms used by Fortune et al. (2014) are suitable for detecting steps in a free-living environment. While multiple accelerometers may be difficult to use in sport, Armitage et al. (2021) recently investigated the reliability of step counting using two accelerometers placed on the right shank. The authors reported excellent inter-unit reliability (intra-class coefficient (ICC) = 0.96) and (95% confidence interval (CI) = 0.90–0.99) during various running-based team sports (Armitage, Beato and McErlain-Naylor, 2021). Additional sport related validation of step counting and identification *via* accelerometry remains necessary.

Different accelerometry derived metrics have been used to quantity external loads and reported in the literature (Bursais et al., 2022). Accelerometry derived external loads have been assessed in field and laboratory environments (Johnstone et al., 2012a; Johnstone et al., 2012b; Johnstone et al., 2012c; Barrett, Midgley and Lovell, 2014). Johnstone et al. (2012a) validated accelerometry-derived training load against oxygen (O_2_) expenditure in a field-based environment and reported a very strong relationship (*r* > 0.90; *p* < 0.01). Accelerometers have also been compared to a heart rate-based measure during soccer training and showed a strong relationship (*r* > 0.80; *p* < 0.01) (Scott et al., 2013). There is a growing body of literature that recognizes the ability of accelerometers to quantify the demands of team and individual sports. Gentles et al. (2018) found strong to nearly perfect correlations between an accelerometry derived training load and session rating of perceived exertion (sRPE) (*r* = 0.84; *p* < 0.001) and total distance measured using GPS (*r* = 0.95; *p* < 0.001) among NCAA women’s soccer players. Accelerometers have also been used to illustrate the differences in the activity profile between single and double match play in tennis (Gentles et al., 2018). In rugby, one study indicated that accelerometers outperformed GPS when quantifying positional (backs vs. forwards) and period (1st vs. 2nd) player movement demands (Howe et al., 2017). Additionally, the within and between device reliability of accelerometers has been established across a variety of movement demands in a laboratory and on-field conditions (Boyd, Ball and Aughey, 2011; Barrett, Midgley and Lovell, 2014; Gómez-Carmona et al., 2019). Two accelerometers aligned on players’ upper back reliably quantified external load (CV 1.9%) during Australian football matches (Boyd, Ball and Aughey, 2011). Furthermore, Gomez-Carmona et al. (2019) assessed the within and between device reliability of eight devices mounted at four anatomical locations during a Sport-Specific Aerobic Field Test (SAFT^90^). The authors reported excellent between-device reliability (CV = 2.96%) and excellent values (Pearson *r* = 0.86–0.96; *p* = 0.46–0.98; *t* = 0.01–0.73) for within-device reliability and no statistically significant differences were noted between trials even though replicating human movement patterns and mechanical efforts on multiple occasions is difficult. Current literature suggests that accelerometers may be used to assess external load, although additional research is needed to evaluate the use of accelerometry data to detect events and quantify external load during sport related movement.

Therefore, the purpose of this study was twofold. First, this study aimed to assess the validity of accelerometers to identify step count during several locomotor conditions including walking, jogging, and running. Second, this study sought to assess the inter-device and test-retest reliability of accelerometers to quantify external load while walking, jogging, and running.

## 2 Materials and methods

### 2.1 Experimental approach to the problem

This investigation was conducted to assess the validity and reliability of a tri-axial accelerometer to identify step count and quantify external load while completing a 20-m straight-line course. Video recording was conducted and served as a reference instrument to evaluate step counts for construct validity.

### 2.2 Participants

Thirty participants (height = 176.8 ± 6.1 cm, weight = 82.3 ± 12.8 kg, age = 26.8 ± 3.1) volunteered to participate in this study. Participants were college students who participated in organized physical activity/exercise at least 3 days a week. This study was approved by the Institutional Review Board of East Tennessee State University and participants provided written consent for their involvement and video recording (IRB#: 0719.16s).

### 2.3 Procedures

**FIGURE 1:**
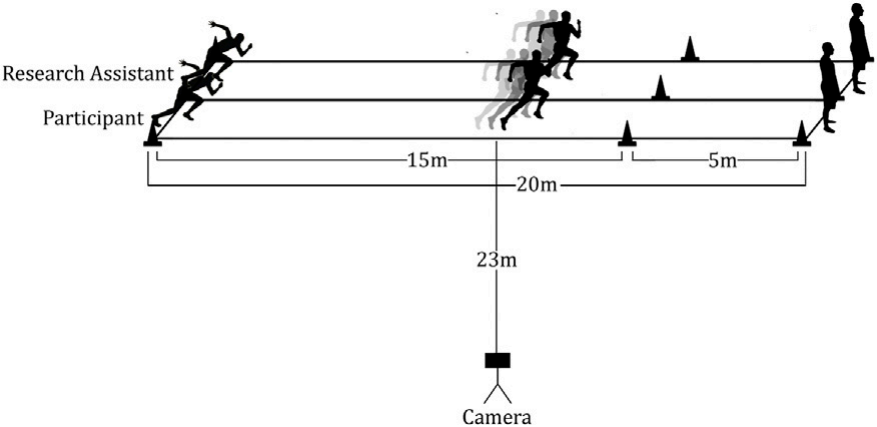
Illustration of The Course Design. *Each participant performed 2 trials × 20 m walking, jogging, and running. *5 m is a deceleration zone for the running course.

The 20-m straight-line course was designed on a grass field (Figure 1). Each participant completed six trials of the experiment. The six trials included two trials of each of the following locomotor conditions: walking, jogging, and running. A research assistant served as a pacemaker in each locomotor condition. A metronome (Pro Metronome App, 2014 EUMLab, Xanin Tech) was used to gait the velocity of each condition (walking, jogging, running) to limit the variation between subjects and trials. The research assistant was wearing headphones to hear the metronome, and participants were directed to keep pace with the research assistant. The tempo was set at 45, 70, and 90 beats per minute for walking, jogging, and running, respectively. A full gait cycle (i.e., two steps) was complete for each beat and a single research assistant guided all trials. The average speed was about 1.0 m/s for walking, 1.8 m/s for jogging, and 4.0 m/s for running. Before initiating each trial, participants were directed to remain still after positioning their feet precisely at the start position; they were also instructed to stop the precisely at the end of the course. Additional signs were placed at 15 m to alert subjects to decelerate in the running course to allow for a precise stop at the finish line. Small stutter steps were sometimes used by subjects to break, particularly during the jogging and running trials, as they approach the end of the course. To keep step counts consistent with the locomotor condition, stutter steps were removed from analysis. Each trial was preceded by a 5 s countdown followed by the command of “go” from the research assistant. Participants performed a familiarization trial for each condition. Following familiarization, each subject completed each condition twice on the 20 m course.

### 2.4 Instrumentation

Acceleration data was collected during each 20 m trial *via* two tri-axial accelerometers sampling at 100 Hz (Zephyr™ BioHarness v3, Zephyr Technology Corp, Annapolis, MD, United States). Two accelerometry derived loads were assessed; 1) Impulse Load (IL) an accumulative measure of mechanical load defined in Table 1 and expressed in arbitrary units, and 2) the square root of the sum of squared accelerations (MAG) expressed as gravitational equivalents (1 g = 9.81 m/s_2_). It should also be noted that IL aims to include only accelerations from locomotor events (e.g., walking, running, jumping) and impacts, but as a proprietary metric, the methods used to identify accelerations from these events are not public.

**TABLE 1:** Formula for each accelerometry based metric.

Metric	Definition and formula^a^
Impulse load^b^	$IL=\circ \sum_{s=1}^{n}\frac{\sqrt{x_{s}^{2}+y_{s}^{2}+z_{s}^{2}}}{9.8067}$
MAG	$MAG=\sum_{s=1}^{n}\sqrt{x_{s}^{2}+y_{s}^{2}+z_{s}^{2}}$

a In the formulas above, x = forward and backward acceleration, y = lateral acceleration and z = vertical acceleration.

b IL, is propriety by the manufacture and is only associated with locomotor events that are detected by Zephyr (e.g., walking, running, bounding, jumping).

Each subject wore two Bioharness™ devices (A and B) located closely together at the xiphoid process level, along the midsternal line. Device A was used for the validity and test-retest reliability, while devices A and B were used to assess inter-device reliability. The beginning and end of each trial were marked by the subject tapping on the accelerometers four times; this served as identifier to expedite data analysis. Video of each trial was recorded for the purposes of step identification using a smartphone camera (iPhone 6; 1080p at 30 fps) and was placed 23 m to the side of the 20 m course (Figure 1).

### 2.5 Event detection validity

**FIGURE 2:**
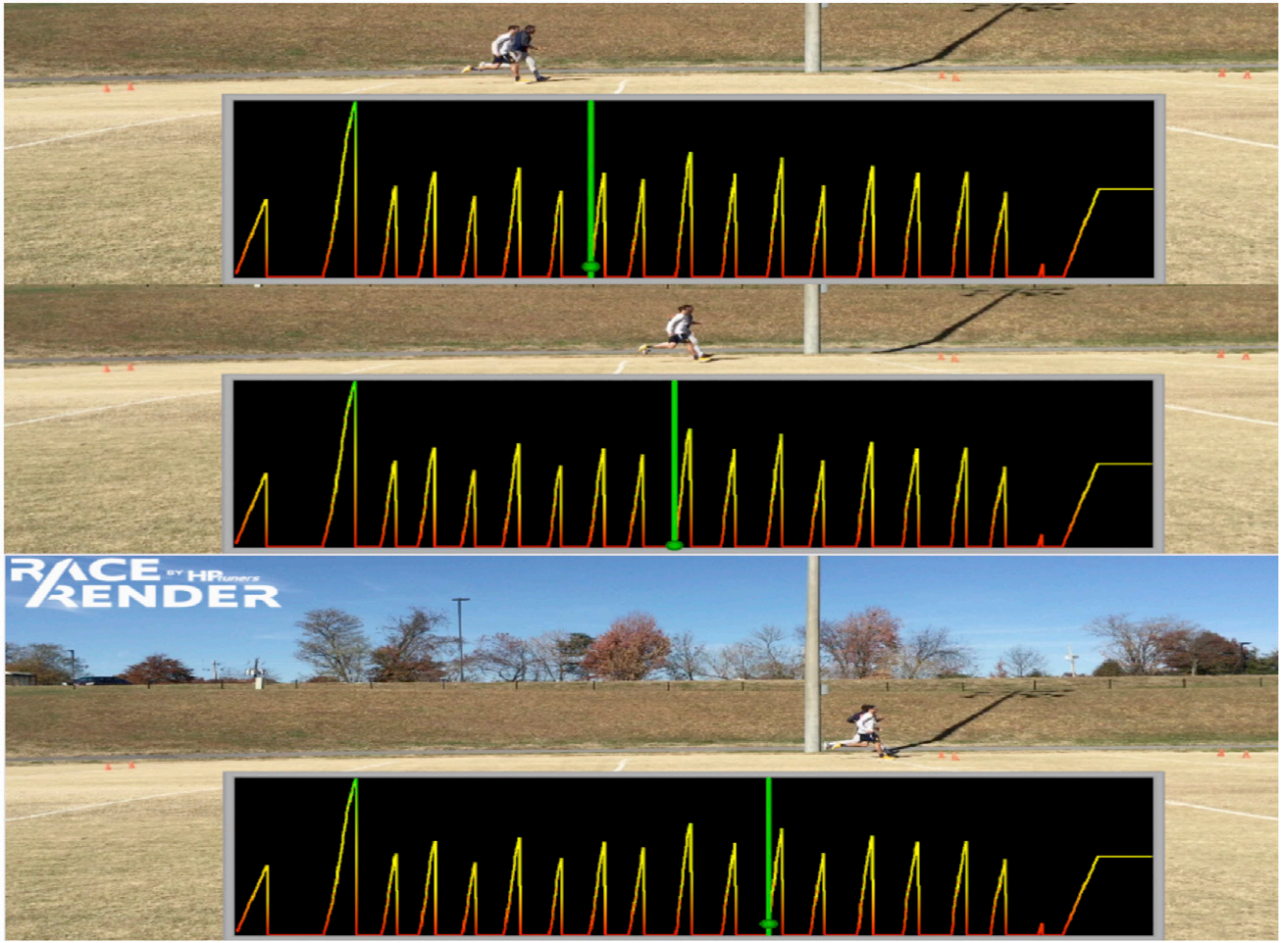
Sequential images and sparklines on each panel represent acceleration data during a running trial. *Video and acceleration data were uploaded to and synchronized using RaceRender software. *Sequential images show the research assistant (far side) and a participant (near side) performing a running trial. *Sparklines represent acceleration data, the initiation of a spike is an onset of a new acceleration and was associated with heel strikes and toe-off on videos to count steps.

Device A was used to assess the ability of the Bioharness™ to detect steps during each locomotion condition. The methods used by Zephyr™ to identify step count detected using the Bioharness™ are proprietary and therefore, we are not able to detail those methods here. Video recording and data from device A were uploaded to and synchronized using RaceRender software (version 3.7.3; 2019 HP Tuners LLC/RaceRender LLC, United States) to identify step count (Figure 2). Heel strike and toe-off were determined using video and associated acceleration data according to step classification recommended from a previous investigation (Pham et al., 2017).

### 2.6 Test-retest and inter-device reliability

Device A was used to assess the test-retest reliability of the Bioharness™ during each locomotor condition. IL and MAG from the first and second trials were assessed. Devices A and B were used to assess the inter-device reliability of the Bioharness™ during each locomotor condition. IL and MAG from the first trial recorded by each device were used for analysis.

### 2.7 Statistical analysis

Accelerometry data were downloaded to OmniSense™ Analysis (version 4.1.4; Zephyr Technology Corporation, Annapolis, MD, United States), then exported to Microsoft Excel 2019 (Microsoft Corporation, Redmond, WA, United States) for analysis. Data were expressed as means and standard deviations for each locomotor condition.

#### 2.7.1 Validity

Agreement and positive predictive value (PPV) were calculated for all trials to assess Bioharness™’s ability to detect steps. Agreement is the percentage of steps detected by device A relative to those counted manually from video. PPV is the ratio of true-positive steps to the sum of true- and false-positive steps. A true-positive step is defined as step identified on video, and identified by device A, while a false-positive step is defined as a step identified by device A, but not identified using video. Bland-Altman plots were also generated to identify systematic error and produce upper and lower limits of agreement between video and device derived methods of step detection (Bland and Altman, 1986).

#### 2.7.2 Reliability

Using the first and second trials of each locomotor condition, test-retest reliability was assessed by calculating the CV and 90% CI for IL and MAG from device A. Additionally, using the first trial from each locomotor condition, inter-device reliability was assessed by calculating CV and 90% CI for IL and MAG from devices A and B. In sports literatures, CV has been categorized as good (<5%), moderate (5%–10%), or poor (>10%) for reliability investigations (Atkinson and Nevill, 1998; Hopkins, 2000; Duthie, Pyne and Hooper, 2003; Currell and Jeukendrup, 2008; Scott, Scott and Kelly, 2016).

## 3 Results

Thirty participants completed a total of 180 trials, 60 trials for each locomotor condition. Means and standard deviations for each metric and trial are detailed in Table 2.

**TABLE 2:** Means and 90% CI for IL, MAG, and step counts for device A and B of trial 1–2.

	Device B		Device A		Device A	
	Trials 1	(90% CI)	Trials 1	(90% CI)	Trials 2	(90% CI)
	Mean		Mean		Mean	
IL						
Walk	77.2 ± 12.1	73.5–80.9	77.0 ± 12.6	73.1–80.9	76.9 ± 11.8	73.2–80.5
Jog	91.4 ± 9.6	88.5–94.4	91.1 ± 9.4	88.2–94.0	90.0 ± 10.7	86.7–93.3
Run	53.7 ± 4.9	52.2–55.3	53.9 ± 5.2	52.3–55.5	53.2 ± 3.6	52.1–54.3
MAG						
Walk	44.8 ± 3.1	43.9–45.8	44.4 ± 3.4	43.3–45.5	45.2 ± 2.3	44.5–45.9
Jog	67.9 ± 9.3	65.1–70.8	67.2 ± 9.5	64.3–70.2	67.5 ± 11.4	64.0–71.1
Run	60.7 ± 8.5	58.0–63.3	60.4 ± 8.3	57.8–62.9	61.1 ± 10.4	57.9–64.3
Video steps						
Walk	—	—	31.5 ± 1.4	31.1–31.9	31.4 ± 1.3	31.0–31.8
Jog	—	—	25.4 ± 2.5	24.6–26.1	25.2 ± 2.6	24.4–26.0
Run	—	—	16.8 ± 1.0	16.5–17.1	16.7 ± 1	16.4–17.0
Bioharness^TM^ steps						
Walk	—	—	31.2 ± 2.5	30.5–32.0	31.1 ± 1.7	30.6–31.6
Jog	—	—	26.0 ± 2.5	25.3–26.8	25.8 ± 2.7	25.0–26.7
Run	—	—	17.9 ± 1.3	16.5–18.3	17.4 ± 1.3	17.0–17.8

IL, impulse load; MAG, magnitude g; CI, confidence interval.

### 3.1 Validity

Bioharness™ demonstrated a PPV of 96.98%–99.41% and an agreement of 93.08%–96.29% in detecting steps during all conditions. The results of each locomotor are detailed in Table 3.

**TABLE 3:** The validity of Bioharness™ in detecting steps.

	Video steps	Device A steps	Percent difference	PPV (%)	Agreement (%)
Walk	1887	1870	−0.9%	96.98	94.97
Jog	1,516	1,556	2.64%	99.41	96.29
Run	1,006	1,058	5.17%	98.91	93.08

PPV, positive predictive value.

**FIGURE 3:**
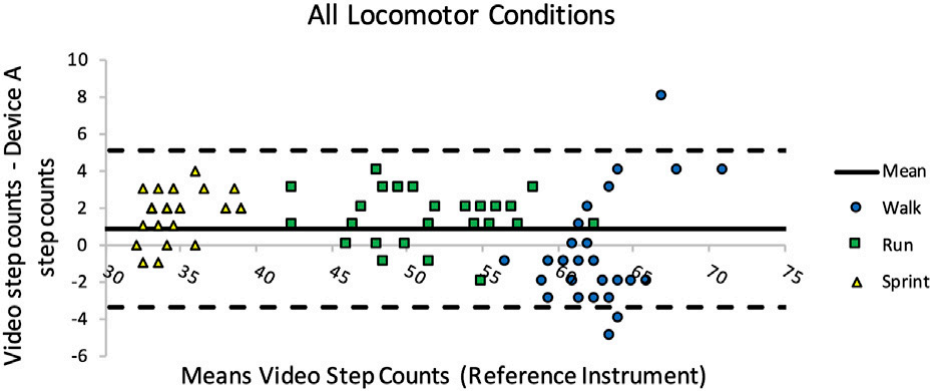
Bland-Altman plot demonstrating the difference between the Bioharness™ and visual step count. *The number of steps taken changes as a result of changes in each locomotor condition velocity. *The solid line is the mean, while the dashed lines represent the repeatability coefficient (±1.96 SD).

Additionally, no systematic error was identifiable using the Bland-Altman plot of Bioharness™ steps counts for all trials as illustrated in Figure 3.

### 3.2 Reliability

Bioharness™ reliability quantified the external load during all courses Both metrics (IL and MAG) demonstrated good reliability between repeated trials and between devices as the CV were below <5% for all conditions. The results of both metrics during all courses are detailed in Table 4.

**TABLE 4:** The test-retest and inter-device reliability of the accelerometry derived metrics IL and MAG.

	Test-retest CV (%)	CV 90% CI	Inter-device CV (%)	CV 90% CI
IL				
Walk	4.99	3.76–6.22%	2.67	1.98–3.37%
Jog	3.22	2.45–3.99%	1.13	0.81–1.43%
Run	4.54	3.46–5.62%	1.82	1.30–2.34%
All conditions	4.25	3.65–4.85%	1.87	1.55–2.19%
MAG				
Walk	3.46	2.59–4.33%	1.69	1.09–2.28%
Jog	3.12	2.25–3.99%	1.61	1.25–1.98%
Run	3.49	2.54–4.44%	2.10	1.61–2.59%
All conditions	3.36	2.86–3.86%	1.80	1.52–2.07%

IL, impulse load; MAG, magnitude g; CV, coefficient of variation; CI, confidence interval. All conditions = all courses combined (walking, jogging, and running).

## 4 Discussion

The purpose of this study was to assess the validity and reliability of accelerometers in identifying step count and quantify external load during different locomotor conditions. A primary finding is that the Bioharness™ is a valid instrument used to detect steps when movement is completed at a range of speeds. Additionally, Bioharness™ is highly reliable to assess external load when walking, jogging, and running are performed. This may also suggest that accelerometry-derived measures can be used to quantify external loads associated with sport-related training and competition.

While Bioharness™ precisely detected steps during all conditions, steps were best detected during jogging trials (PVV = 99.41%, agreement = 96.29%). It appears the Bioharness™ may marginally underestimate total walking steps and slightly overestimate jogging and running steps (Table 3). During walking trials, the Bioharness™ occasionally did not identify steps upon initiating and ending movement. Specifically for the walking conditioning, acceleration during at the beginning and end of the trials may not be of sufficient magnitude to be identified as a step. In contrast, during high-velocity trials, particularly during running, the Bioharness™ recorded false positive steps, potentially due to trunk movement at the beginning and end of each trial. This appears consistent with previous investigations which found that inaccuracies when detecting steps occur most frequently at the beginning and end of locomotion (Dijkstra et al., 2008; Fortune et al., 2014). Nevertheless, Bland-Altman plots revealed low systematic error during all conditions where is the number of steps detected by the Bioharness™ was like that counted manually from video, indicating a high level of agreement (Figure 3).

Despite the difficulties of repeating a locomotor effort accurately, a metronome to gait the speed of participants to reduce intra- and inter-subject differences between trials, was used in this study. This investigation revealed promising test-retest (IL CV = 3.22%–4.99%; MAG CV = 3.12%–3.49%) and inter-device (IL CV = 1.13%–2.67%; MAG CV = 1.61%–2.10%) reliability during all conditions. In accordance with the present results, our earlier observations demonstrated that MAG had lower intra- and inter-device CV when compared to IL and two other accelerometry-derived metrics when assessing external load while walking (Bursais et al., 2022). Future studies should investigate which accelerometry derived metrics best quantify external load in sport. There appears to be some agreement in the literature that step detection and activity classification accuracy using accelerometers improves with a prolonged activity (Grant et al., 2006; Dijkstra et al., 2008; Fortune et al., 2014). However, the Bioharness™ was precise and reliable when detecting steps and quantifying external load from a short bout of exercise.

Several factors may confound accelerometry-derived measures while quantifying external load, including movement artifact of the device, running economy, and stride properties (Barrett, Midgley and Lovell, 2014; Barrett et al., 2016; Malone et al., 2017). In the current study, the two Bioharness™ devices were placed as close to the manufacture’s recommended position but using two devices simultaneously did not permit placement that followed manufacturer guidelines exactly. In addition to device placement, variation in participant anthropometrics and gait may also influence external load and steps detected during each trial. Despite this, test-retest and inter-device reliability for IL (test-retest CV = 3.22%–4.99%; inter-device CV = 1.13%–2.67%) and MAG (test-retest CV = 3.12%–3.49%; inter-device CV = 1.61%–2.10%) were good (<5%) (see Table 4).

Although this study has successfully demonstrated that the Bioharness™ is a valid and reliable instrument for step detection and evaluation of external load, this study has several limitations. First, while the Bioharness™ devices were placed closely together, they could not be placed in the same position. This may cause movement to be measured differently between devices, albeit the differences are likely trivial. Second, while walking, jogging, and running were performed, other actions such as change of direction, jumping, and impacts, were not included. Therefore, caution should be used when applying the current results to individual and team sports. Third, while efforts were made to ensure that participants performed repeat trials in the same manner each time, locomotor variability in speed, stopping location, stride length, and other variables are inevitable. While the Bioharness™ demonstrated good reliability, these limitations make it difficult to isolate the source of variability. Additionally, concerning the research methods where a research assistant was used to gait the velocity of each condition to limit the variation between subjects and trials, this would only test the algorithm accuracy for a limited range of gait speed. Future research should investigate whether accelerometry-derived measures can accurately detect events (e.g., steps, jumps, kicks, and contact) and quantify external load during various sport-related movements including acceleration, deceleration, and directional change.

## 5 Conclusion

The present research aimed to examine the validity and reliability of accelerometers when identifying events and assessing external load during sport-related movements. PVV and agreement analysis, as well Bland-Altman plots, revealed that steps could be accurately identified using accelerometers during walking, jogging, and running. Additionally, good inter-device and test-retest reliability was found for accelerometry-derived measures of external load when locomoting at a range of speeds. The findings of this study may suggest that accelerometry-derived measures can be used to quantify external loads associated with sports training and competition. However, additional research is needed to investigate the use of this technology to detect sporting events (e.g., contact, jumps, sprinting, kicks) and quantify external loads associated with various sports-related movements (e.g., directional change, shuffling, and backward running), which are considered essential characteristics of match play in many sports.

## Data Availability

The raw data supporting the conclusion of this article will be made available by the authors, without undue reservation.
